# Comparative Study of Pulp Vitality in Primary and Young Permanent Molars in Human Children with Pulse Oximeter and Electric Pulp Tester

**DOI:** 10.5005/jp-journals-10005-1291

**Published:** 2015-08-11

**Authors:** Prinka Shahi, PB Sood, Arun Sharma, Manish Madan, Nishat Shahi, Geetanjali Gandhi

**Affiliations:** Senior Lecturer, Department of Pedodontics, MM College of Dental Sciences and Research, Ambala, Haryana, India; Professor, Department of Pedodontics, PDM Dental College Bahadurgarh Haryana, India; Professor, Department of Pedodontics, Institute of Dental Studies and Technologies, Dental College, Ghaziabad, Uttar Pardesh, India; Professor, Department of Pedodontics, MM College of Dental Sciences and Research, Ambala, Haryana, India; Professor, Department of Pedodontics, MM College of Dental Sciences and Research, Ambala, Haryana, India; Senior Lecturer, Department of Orthodontics, MM College of Dental Sciences and Research, Ambala, Haryana, India

**Keywords:** Electric pulp tester (EPT), Pulse oximeter, True positive (TP), True negative (TN), Vitality.

## Abstract

**Aim and objective:** The purpose of this study was to compare the pulp testing methods (pulse oximetry and electric pulp test) in primary and young permanent teeth of children.

**Materials and methods:** The study included a total of 155 children aged 4 to 15 years. Twenty children formed control group I. Study group included all healthy, 85 primary 2nd molars in group II and 85 permanent 1st molars in group III. Fifty children needing endodontics treatment formed test group IV. The readings were recorded as true positive (TP), false positive (FP), true negative (TN), false negative (FN). Based on this, the sensitivity, specificity, positive predictive value and negative predictive value were calculated for each method. The results were statistically analyzed using Chi-square test.

**Results:** On comparing pulse oximetry with electric pulp test ‘p-value’ was found to be 0.487 and 1.00 for groups 1 and 2 respectively and was statistically not significant. Whereas ‘p-value’ for groups 3 and 4 was < 0.0001 and 0.003 respectively and was statistically highly significant.

**Conclusion:** The present study indicates that pulse oximetry can be used as a routine method for assessing the pulp vitality in primary, young permanent and mature permanent teeth.

**How to cite this article:** Shahi P, Sood PB, Sharma A, Madan M, Shahi N, Gandhi G. Comparative Study of Pulp Vitality in Primary and Young Permanent Molars in Human Children with Pulse Oximeter and Electric Pulp Tester. Int J Clin Pediatr Dent 2015;8(2):94-98.

## INTRODUCTION

The vitality of a pulp is function of vascular supply of the pulp within a tooth, and pulpal circulation is true determinant of pulp vitality. The assessment of pulpal status is a diagnostic challenge in clinical practice. An accurate diagnosis is important to achieve a good treatment outcome. The conventional pulp testing methods include thermal stimulation, electrical or direct dentin stimulation fall short of ideal pulp vitality testing as they indirectly monitor pulp vitality by measuring neural res-ponses.^1^ A new approach to determine vital pulp tissue by optical technology includes dual wavelength spectrophotometry, Pulse oximetry, Laser doppler flowmetry, transmitted light-photoplehtysmography. These tests are noninvasive, objective, painless and are very sensitive to detect pulp blood components or blood flow.^2^

The present *in vivo* study was undertaken to evaluate and compare pulse oximeter and conventional pulp testing method (EPT) to assess pulp vitality in primary 2nd molar and young permanent 1st molar.

## MATERIALS AND METHODS

**Fig. 1:**
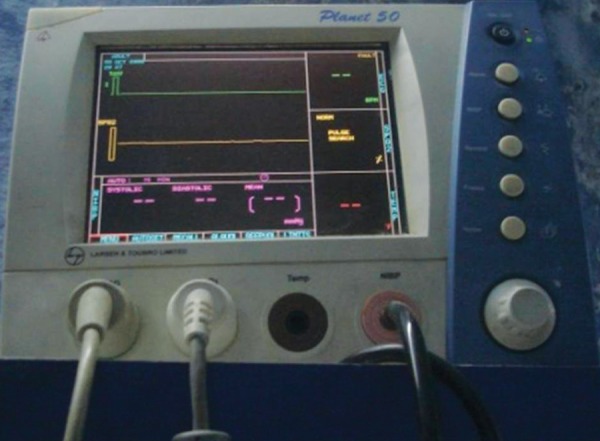
Pulse oximeter

**Fig. 2:**
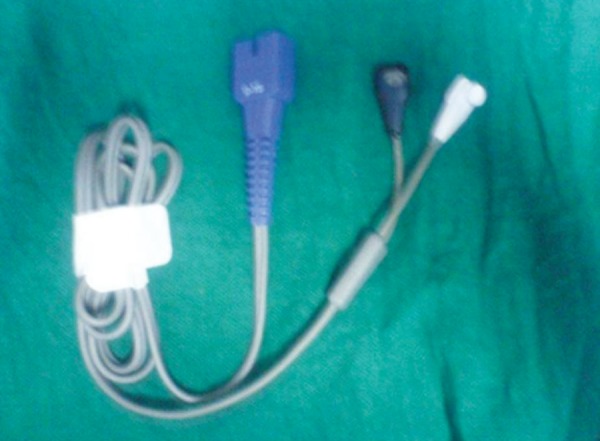
Ear probe

**Fig. 3:**
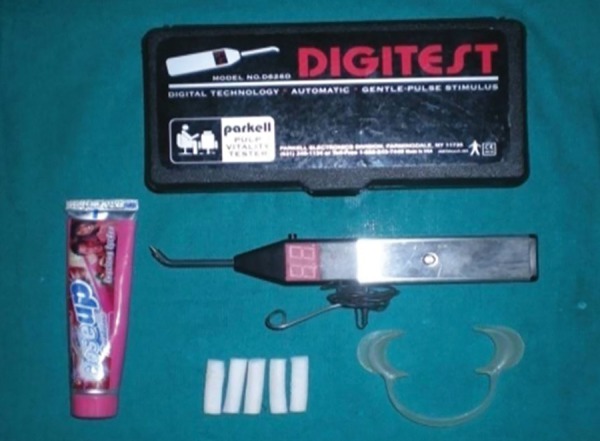
Electric pulp tester

The present *in vivo* study was conducted in the Department of Pedodontics and Preventive Dentistry, ITS– CDSR, Muradnagar, Ghaziabad. Material used: Pulse oximeter with modified ear probe (Nellcor OxiMax) (Figs 1 and 2), Electric Pulp Tester (Gentle-Pulse, Parkell Electronics Division) (Fig. 3), Electrolyte media (toothpaste), cheek retractor, cotton rolls, disposable gloves.

## METHODS OF COHECTION OF DATA

The selection criteria in study group include children that are positively positive on Frankel rating scale between the age group between 6 and 8 years and teeth to be free of caries, restoration, trauma and developmental defect. Patients with any illness (epileptic, mentally challenged), periodontal or periapical pathology in the concerned tooth were excluded.

### Source of Data

One hundred and fifty-five cooperative children aged 4 to 15 years were divided into 4 groups. Group I (Control Group) 20 endodontically treated primary 2nd molars and permanent 1st molars. Group II (Study Group) included all healthy, eighty-five primary 2nd molars. Group III (Study Group) included all healthy, eighty-five young permanent 1st molars. Group IV (Test Group) included 50 teeth in need of endodontic treatment. After completing pulp vitality testing, the pulp chamber in test group teeth were opened for direct visual inspection.

## MATERIALS AND METHODS

**Fig. 4:**
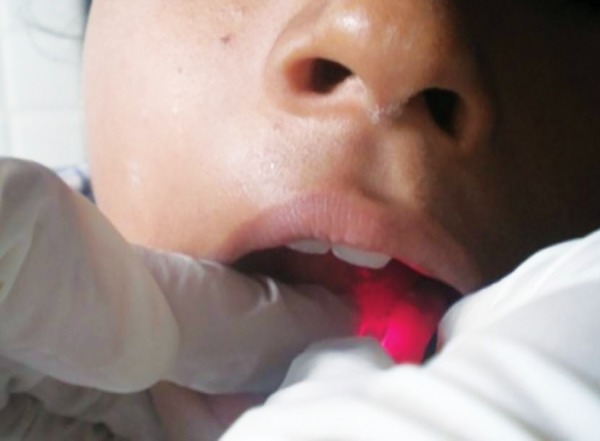
Recording oxygen saturation

**Fig. 5:**
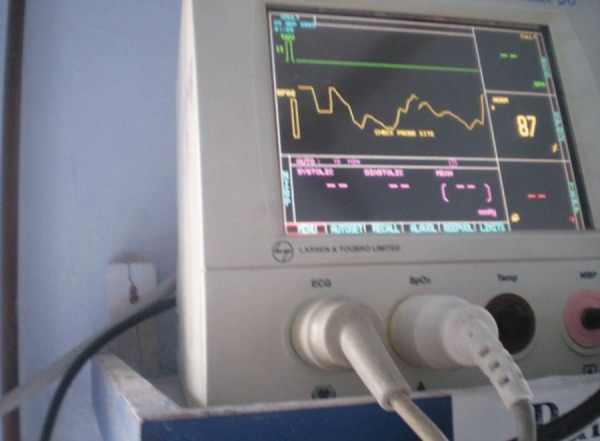
Recording SaO_2_ value

Prior to the study, Ethical clearance and informed consent was taken. Systemic oxygen saturation (SaO_2_) of left index finger was measured first. Thereafter, ear probe of pulse oximetry was firmly placed on buccal surface on middle of the clinical crown for 30 seconds (Figs 4 and 5). Pulse oximetry value above 75% oxygen saturation was taken positive.

**Fig. 6:**
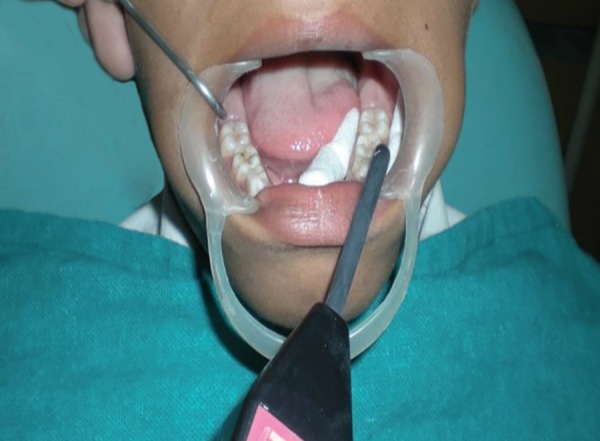
Recording electric pulp tester response

In electric pulp testing tooth was dried and isolated. Electrode was placed on middle of the clinical crown after toothpaste application^3^ (Fig. 6). The response at any level was scored as positive. No response to electrical test was scored as negative. Gloves were not worn during the test.

The readings were recorded as true positive (TP), false positive (FP), true negative (TN), false negative (FN). Based on this, the sensitivity, specificity, positive predictive value and negative predictive value were calculated for each method. Chi-square test was applied to determine statistical difference between pulse oximetry and electric pulp tester in all the four groups.

## RESULTS

The pulse oximetry test identified all the endodontically treated nonvital teeth as nonvital, in group I. All the vital, primary 2nd molars and young permanent 1st molars were identified as vital, in groups II and III respectively. The pulse oximetry test gave 1 false negative reading, in group IV (Table 1).

**Table 1:** **Table 1:** Distribution of response to pulse oximetry test in groups I to IV

*POxi*		*TP*		*FP*		*FN*		*TN*	
Group I		170		0		0		20	
Group II		85		0		0		10	
Group III		85		0		0		10	
Group IV		39		0		1		10	

(TP: True positive; FP: False positive; FN: False negative; TN: True negative)

**Table 2:** **Table 2:** Distribution of response to electric pulp test in groups I to IV

*EPT*		*TP*		*FP*		*FN*		*TN*	
Group I		153		2		17		18	
Group II		85		1		0		9	
Group III		68		1		17		9	
Group IV		35		5		6		4	

(TP: True positive; FP: False positive; FN: False negative; TN: True negative)

**Table 3:** **Table 3:** Comparison between pulse oximetry and electric pulp test in groups I to IV

*POxi vs EPT*		*p-value*		*Level of significance*	
Group I		0.487		NS	
Group II		1.00		NS	
Group III		< 0.0001		HS	
Group IV		0.003		HS	

(NS: Not significant; HS: Highly significant)

The electric pulp test identified 18 of the 20 endo-dontically treated non-vital teeth as non-vital, whereas 2 endodontically treated teeth gave false positive responses, in group I. All the vital, primary 2nd molars were identified as vital, in group II. Of the 85 vital young permanent 1st molars the electric pulp test identified 85, whereas 17 vital teeth gave false negative responses, in group III. The electric pulp test gave 11 false responses, in group IV (Table 2).

On the basis of these findings, the sensitivity, specificity, positive predictive and negative predictive values, accuracy and level of significance (p-value) were calculated for each method in Tables 3 and 4 respectively.

## DISCUSSION

The assessment of pulpal status accurately is important to achieve a good treatment outcome. All conventional vitality tests determined functioning of the nerves. The nerve response to stimulus may not be the true determinant of the pulp vitality^2,4^ as the life of the pulp tissue is dependent on the blood supply and not on nerve functioning, thus are not indicative of the vitality of a pulp.^4^ Among all the conventional vitality tests, EPT was found to be the relative effective method for checking the vitality of the pulp tissue of a tooth.^5^ Studies by Reynolds^6^ and Petersson^7^ also supported this fact.

The newer pulp testing devices, such as laser Doppler flowmetry, dual wavelength spectrophotometry, pulse oximetry and light photoplethysmography are nonin-vasive, objective and have shown more accurate results by detecting the blood supply of the pulp. The pulse oximeter is an affordable, reliable and easily available equipment for an average dental office.^2^ Hence, these two methods were selected for the study.

All the 20 endodontically treated primary 2nd molars and permanent 1st molars recorded SaO_2_ values of 0% with pulse oximetry. Studies by Goho C^8^ and Gopikrishna^9^ also recorded 0% SaO_2_ values on known endodontically treated primary teeth.

The present study showed that all the healthy 85 primary 2nd molars (Group II) and 85 permanent 1st molars (Group III) provided consistent oxygen saturation readings with pulse oximeter. Studies by Gopikrishna,^9^ Goho C,^8^ Calil^10^ and Gopikrishna^11^ have also shown consistent oxygen saturation reading in all vital teeth with pulse oximeter. This demonstrates the ability of the pulse oximeter to differentiate between vital and non-vital teeth.

**Table 4:** **Table 4:** Comparison of sensitivity, specificity, positive predictive value, negative predictive value and accuracy in groups I to IV

		*POxi*										*EPT*									
*Groups*		*Sensitivity*		*Specificity*		*PPV*		*NPV*		*Accuracy*		*Sensitivity*		*Specificity*		*PPV*		*NPV*		*Accuracy*	
I		1.00		1.00		1.00		1.00		1.00		0.90		0.90		0.98		0.51		0.90	
II		1.00		1.00		1.00		1.00		1.00		1.00		0.90		0.98		1.00		1.00	
III		1.00		1.00		1.00		1.00		1.00		0.80		0.90		0.99		0.35		0.80	
IV		0.98		1.00		1.00		0.91		1.00		0.85		0.44		0.87		0.40		0.78	

(PPV: Positive Predictive Value; NPV: Negative Predictive Value)

Group IV has shown one FN reading with SaO_2_ value of 0%, whereas surgically exposed pulp showed sign of normal bleeding. In a study by Gopikrishna, out of 38 vital teeth in need of endodontic treatment, the pulse oximetry test identified 36, whereas two of the vital pulps did not show SaO_2_ to pulse oximetry.^1^ Motion artefact or non-parallelism of probes have been suggested as possible reasons for false reading (Kahan).^12^

In electric pulp testing group 1 showed 10% of false response. Whereas, group II gave 100% positive responses. In young permanent 1st molars EPT showed 20% false responses. Reason for false negative responses in young permanent 1st molars could be―the increased electrometric threshold value in teeth with incomplete root formation, and neural development is incomplete in immature teeth.^13^

Total of 11 out of 50 affected due to disease/trauma showed false responses with EPT. EPT has shown 22% failure. Gopikrishna has found a failure of 18.7% and Petersson has found 18.6% on diseased teeth with EPT. False responses with electric pulp testing could be―traumatized teeth, teeth with incomplete root development, teeth undergone orthodontic tooth movement, pulp canal calcifications. Or the peridental membrane response because of conduction along the moist surface of the tooth, multirooted teeth, patient’s anxiety.^14,15^

In group I (endodontically treated teeth), the sensitivity of pulse oximetry and electric pulp test was 1.00 and 0.90 respectively. The sensitivity of pulse oximetry and electric pulp test were 1.00 in group II (mature teeth). In group III (immature teeth) sensitivity were 1.00 and 0.90 respectively with pulse oximetry and electric pulp test and 0.98 and 0.85 respectively with pulse oximetry and electric pulp test in group IV (diseased teeth). The sensitivity of the pulse oximeter in a study of Gopikrishna^1^ was found to be 1.00, as compared to 0.71 with the electrical test on diseased teeth.

In the present study, the specificity of the pulse oximetry and electric pulp test was 1.00 and 0.90 for groups I, II and III. The specificity of the pulse oximetry and electric pulp test in group IV were 1.00 and 0.44 respectively. The specificity of the pulse oximeter in a study of Gopikrishna^15^ was found to be 0.95, as compared to 0.92 with the electrical test on diseased teeth.

Goho C^8^ evaluated pulp vitality in primary and immature permanent teeth using pulse oximeter and found both sensitivity and specificity to be 1.00. Shender^16^ has concluded the same results with pulse oximeter from his review.

Consistent pulse oximeter readings in this study confirm that pulp circulation and blood oxygen saturation can be detected by pulse oximeter and may be used as a standard and as an indicator of pulp vitality irrespective of the health of tooth, the status of development of tooth, type of dentition and status of disease. Electric pulp testing has a reasonable reliability in primary dentition to evaluate its status of health but it has not been found effective in conditions where the pulp is affected by either a disease process or trauma. Thus, pulse oximeter can be used as an effective diagnostic aid in clinical practice.

## CONCLUSION

This study shows that pulse oximeter is an objective, very sensitive and noninvasive method that can be used as a routine method for assessing the pulp vitality in primary, young permanent and mature permanent teeth. Furthermore, an accurate and immediate diagnosis of pulpal status in traumatized teeth is possible with pulse oximetry. There is a need to evolve the data to suggest saturated oxygen value of the pulpal tissue in terms of percentage as an indicator of the health or disease of the pulp.

## References

[1] Gopikrishna V, Tinagupta K, Kandaswamy D (2007). Evaluation of efficacy of a new custom-made pulse oximeter dental probe in comparison with the electrical and thermal tests for assessing pulp vitality.. J Endod.

[2] Samraj RV, Indra R, Srinivasan MR, Kumar A (2003). Recent advances in pulp vitality testing.. Endodontology.

[3] Kolbinson DA, Teplitsky PE (1988). Electric pulp testing with examination gloves.. Oral Surg Oral Med Oral Pathol.

[4] Bhaskar SN, Rappaport HW (1973). Dental vitality tests and pulp status.. J Am Dent Assoc.

[5] Degering CI (1962). Physiologic evaluation of dental-pulp testing methods.. J Dent Res.

[6] Reynolds RL (1966). The determination of pulp vitality by means of thermal and electrical stimuli.. Oral Surg Oral Med Oral Pathol.

[7] Petersson K, Soderstrom C, Kiani-Anaraki M, Levy G (1999). Evaluation of the ability of thermal and electrical tests to register pulp vitality.. Endod Dent Traum.

[8] Goho C (1999). Pulse oximetry evaluation of vitality in primary and immature permanent teeth.. Pediatr Dent.

[9] Gopikrishna V, Kandaswamy D, Tinagupta . (2006). Assessment of the efficacy of an indigenously developed pulse oximeter dental sensor holder for pulp vitality testing.. Ind J Dent Res.

[10] Calil E, Caldeira CL, Gavini G, Lemos EM (2008). Determination of pulp vitality in vivo with pulse oximetry.. Int Endod J.

[11] Gopikrishna V, Tinagupta, Kandaswamy D (2007). Comparison of electrical, thermal and pulse oximetry methods for assessing pulp vitality in recently traumatized teeth.. J Endod.

[12] Kahan RS, Gulabivala K, Snook M, Setechell DJ (1996). Evaluation of a pulse oximeter and customized probe for pulp vitality testing.. J Endod.

[13] Johnsen DC, Harshbarger J, Rymer HD (1983). Quantitative assessment of neural development in human premolars.. Anat Rec.

[14] Chen E, Abbott PV (2009). Dental pulp testing: a review.. Int J Dent.

[15] Gopikrishna V, Pradeep G, Venkateshbabu N (2009). Assessment of pulp vitality: a review.. Int J Paedtr Dent.

[16] Shender O, Shora S, Siddiqui S, Tchaouchev N, Termei R, Terzioglu U, Toor N, Vishwanath S (2007). How helpful are diagnostic tests for pulp conditions?.

